# Characterization and optimization of heroin hapten-BSA conjugates: method development for the synthesis of reproducible hapten-based vaccines

**DOI:** 10.1007/s00216-014-8035-x

**Published:** 2014-08-02

**Authors:** Oscar B. Torres, Rashmi Jalah, Kenner C. Rice, Fuying Li, Joshua F. G. Antoline, Malliga R. Iyer, Arthur E. Jacobson, Mohamed Nazim Boutaghou, Carl R. Alving, Gary R. Matyas

**Affiliations:** 1 Laboratory of Adjuvant and Antigen Research, US Military HIV Research Program, Walter Reed Army Institute of Research, 503 Robert Grant Avenue, Silver Spring, MD 20910 USA; 2 Department of Health and Human Services, Drug Design and Synthesis Section, Chemical Biology Research Branch, National Institute on Drug Abuse, National Institutes of Health, 9800 Medical Drive, Bethesda, MD 20892-9415 USA; 3 National Institute on Alcohol Abuse and Alcoholism, National Institutes of Health, 9800 Medical Drive, Bethesda, MD 20892-9415 USA; 4 US Military HIV Research Program, Henry M. Jackson Foundation for the Advancement of Military Medicine, 6720A Rockledge Drive, Bethesda, MD 20817 USA; 5 Shimadzu Scientific Instrument, 7102 Riverwood Drive, Columbia, MD 21046 USA

**Keywords:** Hapten density, Drugs of abuse vaccines, TNBS, Ellman’s test, MALDI-TOF MS, ELISA

## Abstract

A potential new treatment for drug addiction is immunization with vaccines that induce antibodies that can abrogate the addictive effects of the drug of abuse. One of the challenges in the development of a vaccine against drugs of abuse is the availability of an optimum procedure that gives reproducible and high yielding hapten-protein conjugates. In this study, a heroin/morphine surrogate hapten (MorHap) was coupled to bovine serum albumin (BSA) using maleimide-thiol chemistry. MorHap-BSA conjugates with 3, 5, 10, 15, 22, 28, and 34 haptens were obtained using different linker and hapten ratios. Using this optimized procedure, MorHap-BSA conjugates were synthesized with highly reproducible results and in high yields. The number of haptens attached to BSA was compared by 2,4,6-trinitrobenzenesulfonic acid (TNBS) assay, modified Ellman’s test and matrix assisted laser desorption ionization time-of-flight mass spectrometry (MALDI-TOF MS). Among the three methods, MALDI-TOF MS discriminated subtle differences in hapten density. The effect of hapten density on enzyme-linked immunosorbent assay (ELISA) performance was evaluated with seven MorHap-BSA conjugates of varying hapten densities, which were used as coating antigens. The highest antibody binding was obtained with MorHap-BSA conjugates containing 3–5 haptens. This is the first report that rigorously analyzes, optimizes and characterizes the conjugation of haptens to proteins that can be used for vaccines against drugs of abuse. The effect of hapten density on the ELISA detection of antibodies against haptens demonstrates the importance of careful characterization of the hapten density by the analytical techniques described.

**Electronic supplementary material:**

The online version of this article (doi:10.1007/s00216-014-8035-x) contains supplementary material, which is available to authorized users.

## Introduction

Addictive drug abuse is a major socially disruptive problem that has a large impact on society and causes a drain on medical, economic, and political systems [1]. There is an estimated 16.5 million people worldwide who use opiates, with the highest prevalence reported in Southwest and Central Asia, Eastern-Southeastern Europe, and North America [2]. Development of vaccines for substance abuse represents a novel approach to conventional therapy. Vaccines to drugs of abuse function by producing circulating antibodies, which bind the drug and prevent it from crossing the blood-brain barrier. This blocks the drug from entering the brain where the drug’s euphoric and addictive effects are induced as a result of interaction with an opiate receptor. Drugs of abuse are small molecules and do not induce antibodies following injection or inhalation. Surrogate compounds (haptens) must be attached to protein molecules (carriers) to be used as immunogens to produce antibodies to the hapten [3, 4]. In this study, MorHap, a heroin/morphine hapten, was used as a model hapten to optimize its attachment to a carrier protein, bovine serum albumin (BSA). Recently, MorHap was attached to tetanus toxoid (TT) and used as a candidate vaccine that induced high titer antibodies, which reacted with heroin and its metabolites, 6-acetylmorphine and morphine, and protected mice from heroin challenge in an antinociception assay [5, 6].

Common carrier proteins that are usually employed in the preparation of hapten-protein conjugates are BSA, diphtheria toxoid (DT), keyhole limpet hemocyanin (KLH), and TT [5–10]. Haptens can be coupled to protein carriers using numerous protein modification strategies [11]. Generally, the choice of conjugation strategy depends on the presence of intrinsic functional groups in the proteins and haptens. The carbodiimide reaction is a popular conjugation strategy for introducing haptens on carriers because surface lysines are ubiquitous in proteins. Carbodiimide chemistry, however, is prone to promote oligomerization due to intermolecular reactions of surface lysines and glutamate/aspartate groups. Protein oligomers are usually insoluble in aqueous solutions and are difficult to characterize. The maleimide-thiol chemistry was employed to circumvent the issue of oligomerization [12]. This coupling chemistry utilizes a heterobifunctional linker with N-hydroxysuccinimide (NHS) ester on one end for coupling to surface lysines and maleimide functionality on the other end for coupling to thiols. Unlike carbodiimide, the maleimide-thiol chemistry tends not to yield higher protein aggregates because thiols in proteins are predominantly in an oxidized state and cysteine residues are less abundant in proteins compared to lysine residues [13]. Recently, thiolated haptens and maleimide-conjugated proteins as carriers are becoming more frequently used for the synthesis of vaccines against drugs of abuse [5–7, 9, 10].

In general, the chemical structure of the hapten and the hapten density (number of covalently attached haptens per molecule of the carrier protein) are critical for the generation of effective antibody titers. For heroin vaccines, heroin-like haptens with varying stabilities at physiological pH and morphine-like haptens have been reported [5, 6, 9]. MorHap, a heroin/morphine-like hapten, is particularly interesting because it has been shown to induce high antibody titers [5, 6]. Recently, the effect of hapten density on the immune response has been demonstrated for a methamphetamine vaccine [7], but the effect of hapten density on the ELISA coating antigen, or for opiate vaccines, has not been studied.

Hapten density has often been mediated by coupling chemistry strategies, stoichiometry of the reacting partners, and the nature of the carrier protein [12]. The hapten density has been assessed by direct and indirect methods. Direct methods have measured changes in the original protein properties, such as UV absorption, fluorescence, and mass. If the hapten is a stronger chromophore than the aromatic residues of the protein, the number of haptens can be measured by changes in absorbance/fluorescence. Since these spectrophotometric techniques do not account for the noncovalent binding of the haptens to the protein, absorbance/fluorescence measurements can overestimate hapten density. Gel electrophoresis and matrix assisted laser desorption ionization time-of-flight mass spectrometry (MALDI-TOF MS) are direct methods of assessing hapten density because they measure changes in mass before and after conjugation [14, 15]. In contrast to MALDI-TOF MS, gel electrophoresis cannot discriminate subtle differences in mass. Quantifying the number of haptens attached to complex proteins by direct methods is a challenging task. Pryde and coworkers used a labor-intensive reversed phase HPLC analysis of acid-hydrolyzed hapten-DT conjugates to measure the number of haptens [8]. Their approach, however, is not suitable for haptens that degrade at low pH. Unsuccessful measurements of hapten density by conventional MALDI-TOF MS have been also reported for heroin/morphine-KLH conjugates [9, 16]. With the latest developments on high mass MALDI-TOF MS, characterization of these complex conjugates might be possible in the future [17–19].

2,4,6-Trinitrobenzene sulfonic acid (TNBS), which reacts with free amines, is a traditional indirect method for assessing hapten density in proteins [20–22]. The amount of surface lysines decreases after conjugation. The difference in the number of amines before and after coupling reactions is correlated to hapten density. The modified Ellman’s test is also an indirect method of measuring hapten density for conjugates that were synthesized using maleimide-thiol coupling chemistry [23]. The modified Ellman’s test measures the amount of maleimide in the activated BSA intermediate. Maleimide number is equivalent to the number of haptens, because there is a 1:1 stoichiometry between maleimide and thiolated haptens [12]. Both the TNBS assay and modified Ellman’s test measure the number of reactive functional groups and are a measure of “hapten equivalences” and not hapten density per se. Therefore, an optimum procedure must be developed to ensure that hapten equivalences correspond to hapten density.

Another consideration for vaccine development that is concomitant with hapten density is the protein yield of the synthesized conjugates. For future use as a licensed vaccine, efforts must be invested to optimize the yield of hapten-protein conjugates. Despite the relevance of hapten density and protein yield in vaccine development, the quantitative relationship between the two components has not been investigated. This is most likely due to the lack of optimized coupling protocol that gives reproducible hapten-protein conjugates. In the present study, the conjugation of MorHap to BSA was optimized using appropriate linker and hapten ratios to BSA. The effect of MorHap density on BSA was tested as a coating antigen in an ELISA to measure antibodies to MorpHap, which yielded the surprising result that highest antibody binding was obtained with the least amount of attached haptens.

## Materials and methods

BSA, used for coupling to MorHap, NHS-(PEG)_2_-maleimide linker (SM-(PEG)_2_), spin desalting column (Zeba, 7K molecular weight cut-off (MWCO)), dialysis cassettes (Slide-A-Lyzer G2, 10K MWCO), bicinchoninic acid (BCA) protein assay kit, phosphate buffered saline (PBS, 100 mM sodium phosphate, 150 mM NaCl, pH 7.2) that was used for the coupling reaction, and ultrapure 10 % sodium dodecyl sulfate (SDS) were purchased from Pierce Protein Research/Thermo Fisher Scientific (Rockford, IL). Trifluoroacetic acid (TFA), triisopropylsilane (TIS), dimethylsulfoxide (DMSO), 5,5′-dithiobis(2-nitrobenzoic acid) (Ellman’s reagent), sodium 2-mercaptoethanesulfonate (MESNA), l-glutamic acid, sinapinic acid, UV-transparent 96-well plates, mass spectrometry grade formic acid (FA), BSA that was used as MALDI-TOF calibration standard and BSA that was used as blocking reagent for ELISA were purchased from Sigma-Aldrich (Saint Louis, MO). TNBS was purchased from G-Biosciences (St. Louis, MO). ZipTip (C_4_ resin) was purchased from Millipore (Billerica, MA). Polysulfone membrane filter (0.22 μm) was purchased from Pall Corporation (Port Washington, NY). Immulon™ 2HB flat ELISA plates were from Thermo Scientific (Marietta, OH). Mouse anti-morphine monoclonal antibody BD1263 was purchased from Abcam (Cambridge, MA). Peroxidase-linked sheep anti-mouse IgG (γ-chain specific) was purchased from The Binding Site (San Diego, CA). 2,2′-Azino-di(3-ethylbenzothiazoline-6-sulfonate) (ABTS) peroxidase substrate system was purchased from KPL, Inc. (Gaithersburg, MD). Mass spectrometry grade water and acetonitrile (ACN) were obtained from Fisher Scientific. Dulbecco’s phosphate-buffered saline (DPBS, 10 mM Na_2_HPO_4_, 1.8 mM KH_2_PO_4_, 2.7 mM KCl, 137 mM NaCl, pH 7.4), used for dialysis, was purchased from Quality Biological Inc. (Gaithersburg, MD).

### Deprotection of MorHap

Deprotected hapten was obtained by dissolving trityl-protected MorHap (6.0 mg) in chloroform (656 μL, 87.5 %), treating with TFA (75 μL, 10 %) and TIS (19 μL, 2.5 %) for 1 h at RT, and concentrating the reaction mixture under high vacuum overnight. The crude residue was solubilized in water (1 mL) with sonication and was membrane filtered.

### Coupling procedure described by Matyas et al. [6]

**Fig. 1:**
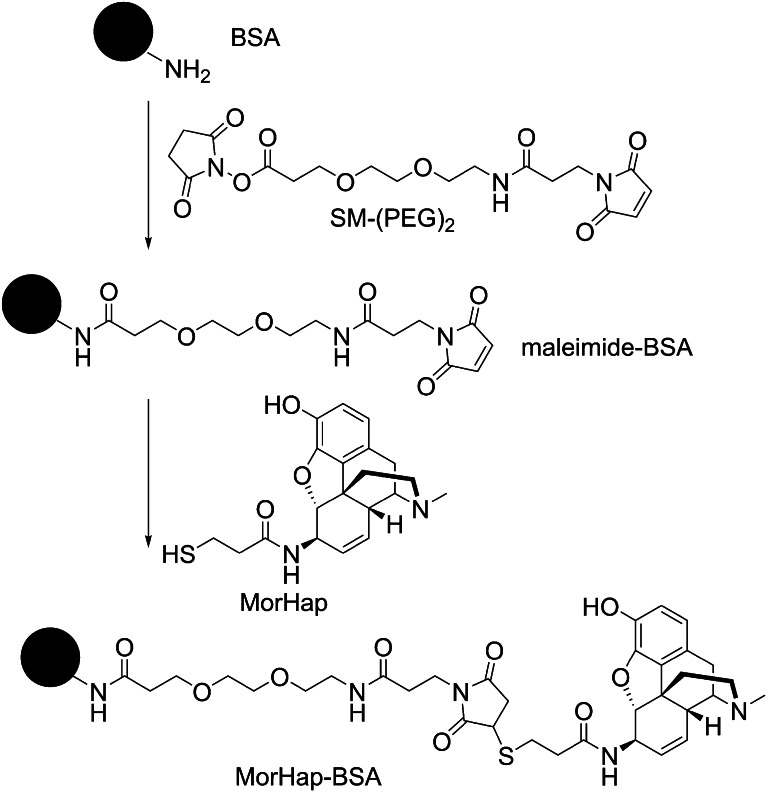
Synthesis of MorHap-BSA conjugates using thiol-maleimide coupling chemistry

SM-(PEG)_2_ linker (6.0 mg) and BSA (2.5 mg) were incubated in 1 mL DPBS. The molar conjugation ratio of the linker to BSA was approximately 400. After stirring for 2 h at RT, excess linker was removed by overnight dialysis in DPBS at 4 °C. An aqueous solution of MorHap (~1 mL) was added dropwise to a stirring solution of dialyzed BSA-(PEG)_2_-maleimide intermediate (maleimide-BSA). Following incubation at RT for 2 h, excess hapten was removed by overnight dialysis DPBS at 4 °C. The BSA-(PEG)_2_-MorHap conjugate (MorHap-BSA) was membrane filtered and the protein concentration was measured by BCA assay. The conjugation route for the preparation of MorHap-BSA conjugates is shown in Fig. 1.

### Optimization of the coupling procedure

SM-(PEG)_2_ linker (60 μL, 250 mM in DMSO) and BSA (2.5 mg, 10 mg/mL in PBS) were incubated in 1 mL PBS for 2 h at RT. The molar conjugation ratio of the linker to BSA was 400. The linker was prepared as a DMSO stock solution to avoid potential error in weighing small amount of reagents. Excess linker was removed by either overnight dialysis against DPBS or spin desalting column.

An aqueous solution of MorHap was prepared as described above, but was purified prior to solubilization in water. Briefly, the trityl side product of hapten deprotection was removed by petroleum ether wash (500 μL, three times). The complete removal of the side product was confirmed by thin layer chromatography. MorHap was added dropwise to a stirring solution of purified maleimide-BSA. Following incubation at RT for 2 h, excess MorHap was removed by either overnight dialysis at 4 °C or spin desalting column. The MorHap-BSA conjugate was membrane filtered and the protein recovery was determined by BCA assay.

### Coupling procedure for the preparation of MorHap-BSA with varying hapten densities

BSA (2.5 mg) was treated with different amounts of SM-(PEG)_2_ linker (0.75, 1.50, 3.75, 7.5, 15, 30, or 60 μL of 250 mM in DMSO) and incubated in 1 mL PBS for 2 h at RT. This corresponded to a linker ratio of 5, 10, 25, 50, 100, 200, and 400, respectively. Excess linker was removed by spin desalting column. Protein aliquots were taken to determine the maleimide content of the activated BSA.

An aqueous solution of MorHap was prepared and washed with petroleum ether as described above. In addition, the amount of thiols in solution was measured by Ellman’s assay. MorHap was added dropwise to a stirring solution of purified maleimide-BSA. The different molar conjugation ratios of the hapten to maleimide-BSA tested were 25, 100, and 400. The volume of the mixture was equalized by adding PBS. Following incubation at RT for 2 h, excess MorHap was removed by overnight dialysis at 4 °C. The MorHap-BSA conjugate was membrane filtered and the protein recovery from maleimide-BSA to MorHap-BSA was determined by BCA assay. The hapten equivalences/density was measured by the TNBS assay and MALDI-TOF MS.

### Ellman’s assay

In the conventional Ellman’s assay, thiol concentration of the sample is measured based on the standard curve of a water-soluble thiol, MESNA. Briefly, a standard curve was prepared by adding the Ellman’s reagent to different concentrations of MESNA. An aliquot of MorHap was diluted in 100 mM sodium phosphate buffer (PB), pH 8.0 (800 μL final volume) and treated with Ellman’s reagent (160 μL, 10 mM in PB, pH 8.0). The absorbance of the reaction mixture was read at 412 nm and the thiol concentration was quantified from the standard calibration curve. The thiol concentration was used to calculate for the molar conjugation ratio of MorHap to maleimide-BSA.

### Modified Ellman’s test

**Fig. 2:**
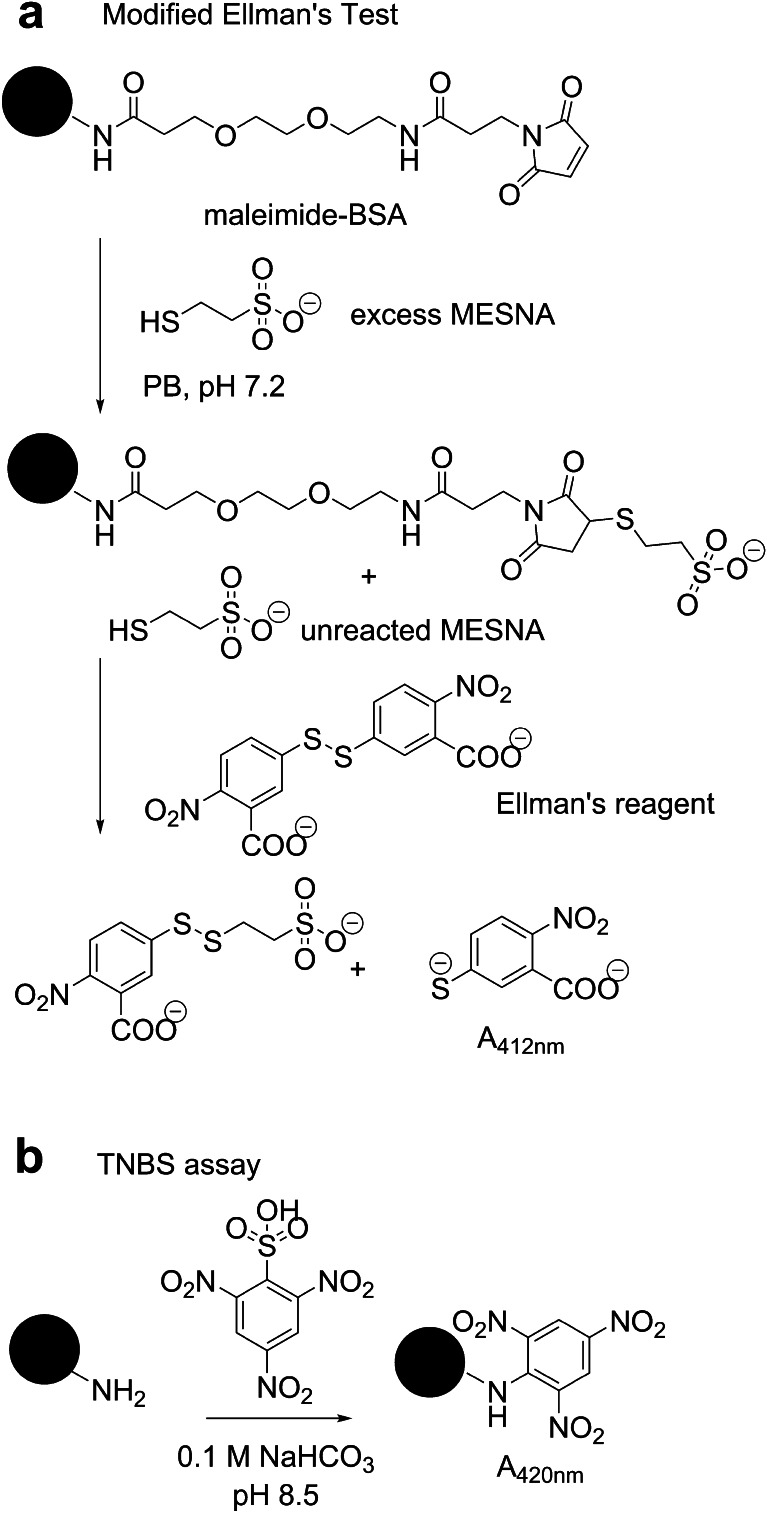
Indirect methods for measuring hapten density of protein conjugates. Modified Ellman’s test was performed by treating maleimide-BSA intermediate with excess MESNA and subsequently back titrating the unreacted MESNA with Ellman’s reagent (**a**). TNBS assay was executed by reacting MorHap-BSA conjugates with TNBS in basic buffer (**b**). Both chemical assays measured hapten equivalences

The modified Ellman’s test was used to measure the concentration of maleimide in the sample. Maleimide-BSA was allowed to react with excess MESNA. After the reaction, unreacted MESNA was back titrated with Ellman’s reagent (Fig. 2a). The difference between the initial and final MESNA concentration corresponded to the maleimide content of the sample. An aliquot of maleimide-BSA was suspended in PB, pH 7.2, and subsequently, reacted with excess MESNA (160 μL, 500 μM in PB, pH 8.0). The final volume and final protein concentrations, [maleimide-BSA], of the reaction mixture were 800 μL and 1 μM, respectively. The reaction mixture was incubated at RT for 5 min. Ellman’s reagent (160 μL, 10 mM in PB, pH 8.0) was added to the reaction and absorbance was measured at 412 nm. The amount of unreacted MESNA, [MESNA]_final_, was determined from a calibration curve. The initial concentration of MESNA, [MESNA]_initial_, was the excess MESNA described above without the protein. The number of maleimides per protein was calculated using the expression:

$$ \mathrm{Number}\ \mathrm{of}\ \mathrm{maleimides} = \frac{\left({\left[\mathrm{MESNA}\right]}_{\mathrm{initial}}-{\left[\mathrm{MESNA}\right]}_{\mathrm{final}}\right)}{\left[\mathrm{maleimide}\hbox{-} \mathrm{BSA}\right]} $$

The number of maleimides represented hapten equivalences since the maleimide group in maleimide-BSA was the reaction partner of MorHap.

### TNBS assay

TNBS specifically reacts with primary amines to give a yellow-colored product that can be monitored at 420 nm (Fig. 2b). MorHap-BSA conjugates were suspended in 0.1 M NaHCO_3_, pH 8.0, and treated with TNBS (250 μL, 0.01 % in 0.1 M NaHCO_3_). The final volume and final protein concentration, [MorHap-BSA], of the reaction mixture were 500 μL and 1 μM, respectively. The reaction was incubated for 2 h at 37 °C and the absorbance was measured at 420 nm. The free amine concentration, [Amine], was calculated from the corresponding calibration curve plotted using l-glutamic acid as a standard. The number of amines per protein was calculated using the expression:

$$ \mathrm{Number}\kern.3em \mathrm{of}\kern.3em \mathrm{amines}=\frac{\left[\mathrm{Amine}\right]}{\left[\mathrm{MorHap}\hbox{-} \mathrm{BSA}\right]} $$

The difference in the number of free amines before and after conjugation represented hapten equivalences because the amino group reacts with the linker during the coupling process.

### MALDI-TOF MS

The mass of the MorHap-BSA conjugates was determined using Axima MegaTOF™ (Shimadzu Scientific Instruments, Columbia, MD). The Axima MegaTOF™ is capable of ultra high mass analysis up to the megadalton level. Briefly, BSA starting material and MorHap-BSA conjugates were desalted using C_4_ ZipTip. The proteins (0.5 μL) were mixed with (0.5 μL) sinapinic acid (10 mg/mL in 50:50 ACN/H_2_O 0.1 % FA) and spotted on a MALDI-TOF 384-well stainless plate. The protein-matrix samples were allowed to crystallize at RT. Prior to measurements, the instrument was calibrated against BSA MALDI-TOF calibration standard. Hence, the ionization of the MorHap-BSA conjugates was expected to be similar and any mass shift was attributed to hapten incorporation into the protein. Mass spectra of BSA starting material and MorHap-BSA conjugates were acquired by averaging 500 mass profiles in the linear mode. Mass spectra were smoothed using the Gaussian method and mass assignments were done using threshold apex peak detection. The number of the haptens was calculated using the expression:

$$ \mathrm{Number}\kern.3em \mathrm{of}\kern.3em \mathrm{haptens}=\frac{{\mathrm{Mass}}_{\mathrm{MorHap}\hbox{-} \mathrm{BSA}}-\kern0.5em {\mathrm{Mass}}_{\mathrm{BSA}}}{{\mathrm{Mass}}_{\mathrm{MorHap}\hbox{-} \mathrm{linker}}} $$

The net mass addition of MorHap and linker, Mass_MorHap-linker_, was 682.27 g/mol.

### ELISA

MorHap-BSA conjugates of varying hapten density (0.1 μg BSA in 100 μL of DPBS) were added to the ELISA plates and incubated at 4 °C overnight. The remainder of the ELISA was performed as described [5, 6]. Briefly, the plates were blocked with 1 % BSA in 20 mM Tris-0.15 M sodium chloride, pH 7.4, for 2 h. Mouse sera and mouse anti-morphine monoclonal antibody were diluted in blocker and added to the plates. Mouse sera were obtained from Balb/c mice immunized with MorHap-TT conjugates from the study of Matyas et al. [6]. Following incubation for 2 h at RT, the plates were washed with 20 mM Tris-0.15 M sodium chloride-0.05 % Tween 20®. Peroxidase-linked sheep anti-mouse IgG diluted in blocker was added and the plates were incubated for 1 h at RT. The plates were washed and ABTS peroxidase substrate system (100 μL) was added. After incubation at RT for 1 h, 1 % SDS (100 μL) was added to stop the reaction and the absorbance was read at 405 nm.

### Data analysis

Statistical analysis was performed using GraphPad Prism. A *T* test (unpaired, one tail) was used for comparison of maleimide content. A one-way ANOVA with Dunn’s correction for multiple comparisons was used to compare the effect of the purification steps on protein yield and the effect of hapten density to ELISA absorbance. A two-way ANOVA with Tukey’s correction for multiple comparisons was used to compare the direct and indirect methods for quantifying hapten density, the effect of MorHap:maleimide-BSA ratios on protein yield, and the effect of MorHap ratios on hapten density.

## Results and discussion

### Optimization of protein yield and number of maleimides

The thiolated MorHap was conjugated to BSA in a two-step reaction using SM-(PEG)_2_. Surface lysines of BSA were first reacted with the NHS ester end of the linker to give an activated maleimide-BSA intermediate. The subsequent step used the Michael addition of MorHap to maleimide end of the BSA intermediate. Using the coupling procedure described by Matyas et al., the MorHap-BSA conjugate was obtained with only a 5–10 % yield [6]. This poor yield may be due to the precipitation of the BSA conjugate due to the addition of unpurified hapten. Since BSA binds fatty acids [24], it was plausible that the binding of the BSA conjugates to the free trityl side product decreased the water solubility of the BSA. When the trityl side product was removed by a petroleum ether wash after the deprotection of MorHap, the yield of the conjugate was improved to 20–25 %.

**Fig. 3:**
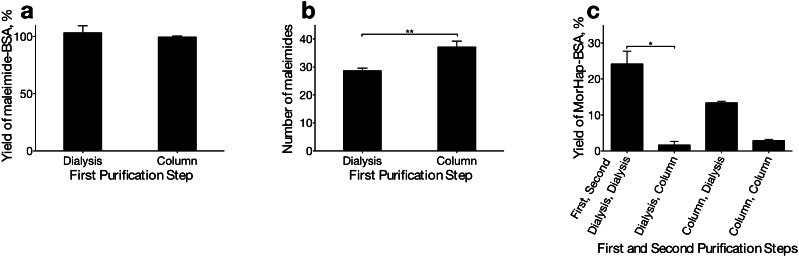
Effect of purification steps on protein yield and maleimide content. BSA was treated with 400-fold molar excess of the SM-(PEG)_2_. Excess linker was removed by either spin desalting column or dialysis (first purification). The protein yield and maleimide content of maleimide-BSA was determined by BCA (**a**) and modified Ellman’s test (**b**), respectively. The number of maleimides statistically decreased after overnight dialysis (**p < 0.01, *T* test). The combined effect of the two purification steps on protein yield was assessed by BCA (**c**). The yield of MorHap-BSA was significantly decreased in the second column purification (*p < 0.05, one-way ANOVA). Values are the mean of three independent experiments ± standard deviation

Purification of protein conjugates has been an important prerequisite in method development [25]. The effects of the two purification steps, purification after linker addition and purification after hapten addition, on the protein yield of the MorHap-BSA were investigated. Briefly, BSA was treated with a 400-fold molar excess of the SM-(PEG)_2_. The protein yield of the maleimide-BSA was not significantly different between the desalting column or dialysis procedures (Fig. 3a). Since the number of maleimides corresponds to the number of haptens attached, the selection of the purification method was not only dependent on the protein yield, but also on the maleimide content of the BSA. Overnight dialysis of the activated BSA led to the loss of approximately 20 % of the maleimide compared to the column-purified maleimide-BSA (Fig. 3b). Although the apparent rate of hydrolysis of maleimide to maleamic acid was slow [26], its cumulative degradation became pronounced over the 14–18 h of dialysis.

The effect of the method of purification on the protein yield after hapten addition was also investigated. The protein yield of MorHap-BSA was significantly decreased when desalting columns were used for purification compared to dialysis (Fig. 3c). The decrease in the protein yield suggested that covalent attachment of the hapten increased the overall hydrophobicity of MorHap-BSA conjugate. This was confirmed by the observation that the maleimide-BSA solutions became cloudy after hapten addition. This suggested that the MorHap-BSA conjugate aggregated and either did not pass through the column resin or also adhered to the column, thereby, reducing the protein yield. A similar loss of protein yield was not observed in the column purification of maleimide-BSA because the decrease in the positive charge of BSA after linker conjugation was compensated by the introduction of the hydrophilic PEG chain. Based on the protein yield and the number of maleimides, the column and dialysis purification methods were chosen as the first and second purification procedure, respectively. Furthermore, the combination of the two purification procedures decreased the time of the overall synthesis procedure from 3 to 2 days.

Our initial work on optimization suggested that the number of haptens covalently attached to BSA and the concentration of unreacted haptens in solution may influence protein yield. Since MorHap is a hydrophobic hapten, BSA with 35 MorHap molecules attached was less soluble in water than BSA with fewer haptens. The solubility of BSA with 35 MorHap molecules attached also may be different in the presence of excess unreacted haptens. The coupling procedure was further explored by examining the effects of hapten density and excess hapten in solution on protein yield.

### Molar ratio of linker to BSA dictates hapten density

**Fig. 4:**
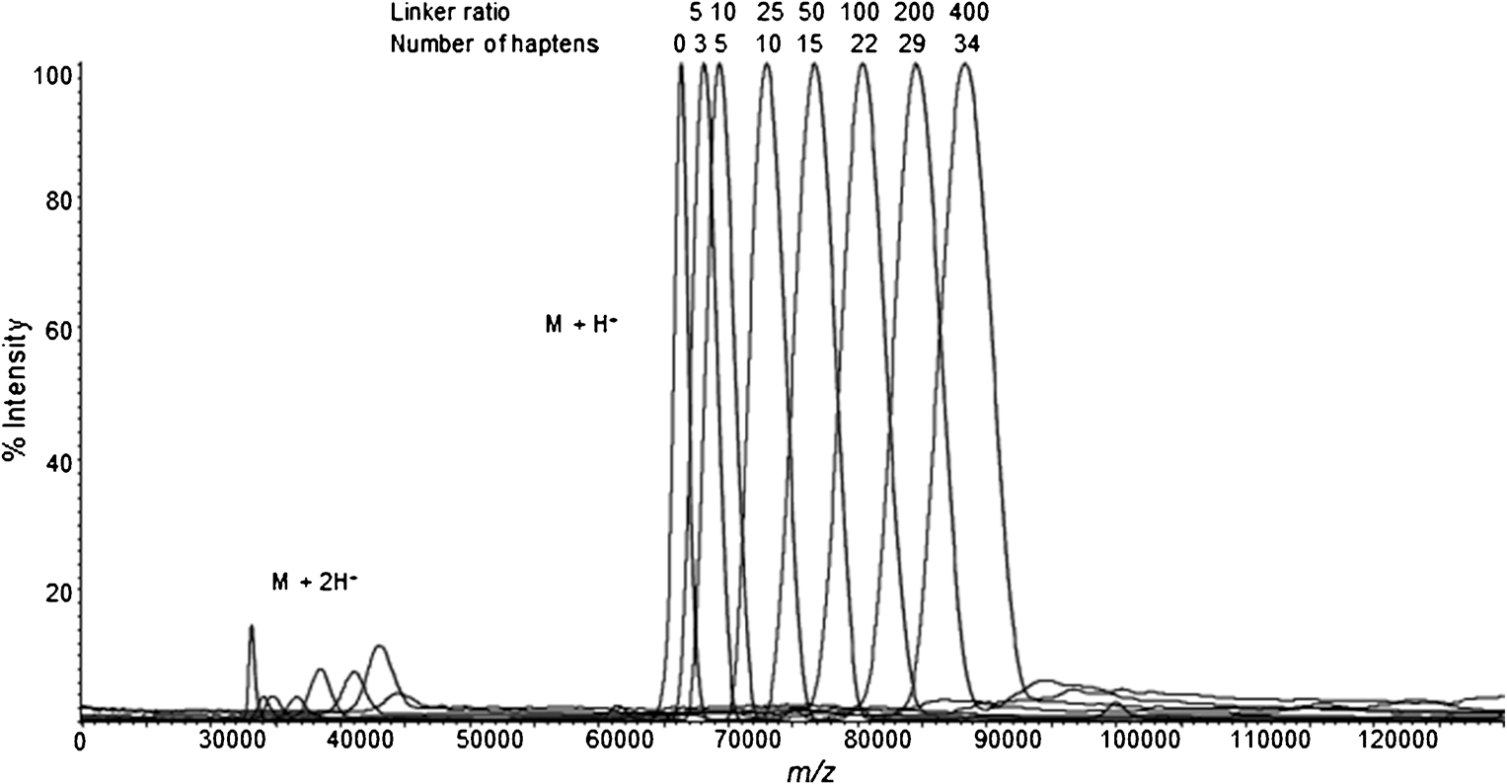
MALDI-TOF mass spectra of MorHap-BSA conjugates with varying hapten densities. The representative MALDI-TOF mass spectra for the seven MorHap-BSA conjugates were overlaid with the mass spectrum of unreacted BSA. The number of haptens attached to BSA was directly proportional to linker:BSA molar ratios

To accurately dissect the relationship between hapten density and protein yield, MorHap-BSA with varying hapten densities was synthesized. Briefly, BSA was treated with an increasing molar conjugation ratio of SM-(PEG)_2_ linker: 5, 10, 25, 50, 100, 200, and 400. After column purification, the maleimide-BSA intermediates were treated with 400-fold molar excess of MorHap. Precipitates were not observed when MorHap was added to maleimide-BSA derived from 5, 10, 25, 50, and 100 molar ratio reactions. In contrast, an opaque mixture and solid aggregates were observed for 200 and 400 molar ratio reactions, respectively. Thus, MorHap-BSA conjugates with low hapten densities were more soluble in aqueous environment than MorHap-BSA conjugates with high hapten densities. To verify if these observations were due to an increase in hapten density, the masses of the dialyzed conjugates were measured by MALDI-TOF MS. Mass spectra overlays revealed distinct peaks for each molar conjugation ratio (Fig. 4). Using MALDI-TOF MS, M + H^+^ and M + H^2+^ for each unique conjugates were detected in acceptable relative intensities. Moreover, no oligomeric forms of MorHap-BSA conjugates were observed in the mass spectra. Since all M + H^+^ peaks exhibited Gaussian profiles, accurate mass measurements can be made for the MorHap conjugates [27]. MorHap-BSA conjugates with 3, 5, 10, 15, 22, 28, and 34 haptens were obtained using linker to BSA ratios of 5, 10, 25, 50, 100, 200, and 400, respectively. Although hapten density is dependent on the molar ratio of linker to BSA, the relationship between the two was not linear. For example, the linker ratio of 25 did not translate to 25 haptens due to steric hindrance associated with the coupling reaction and the inherent reactivity of SM-(PEG)_2_ in aqueous buffer. At physiological pH, water and surface lysines compete for the NHS ester end of the linker, while the maleimide end hydrolyzes to maleamic acid. Thus, the actual hapten density must be determined empirically for any given linker to BSA ratios.

### Comparison of methods for quantifying hapten density: MALDI-TOF MS, TNBS assay, and modified Ellman’s test

**Fig. 5:**
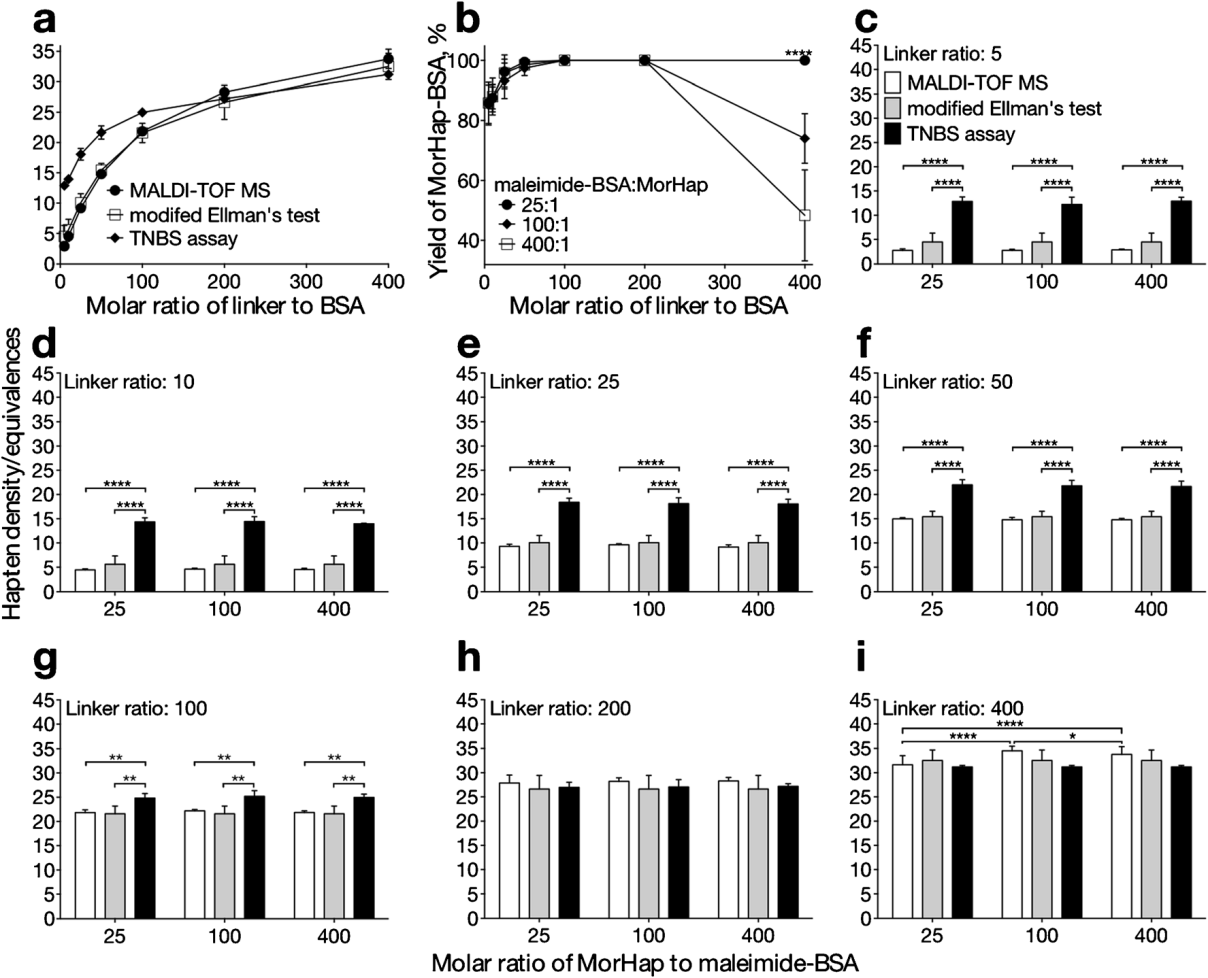
Effect of linker and hapten ratios on hapten density and protein yield. Comparison of direct and indirect methods for quantifying hapten density/equivalences (**a**). There was no significant difference in the number of haptens quantified by modified Ellman’s test and MALDI-TOF MS in all linker:BSA conjugation ratios. TNBS overestimated the number of haptens at 5, 10, 25, 50 (*p* < 0.0001, two-way ANOVA), and 100 (*p* < 0.01, two-way ANOVA) linker:BSA molar conjugation ratios. Effect of hapten ratios on the yield of MorHap-BSA conjugates (**b**). The protein yield was significantly different at linker:BSA molar ratio of 400 (****p < 0.0001, two-way ANOVA). Effect of hapten ratios on the hapten density of MorHap-BSA conjugates (**c**–**i**). TNBS overestimated the number of haptens at low linker:BSA conjugation ratios similar to Fig 5a (**p < 0.01 and ****p < 0.0001, two-way ANOVA). There was no significant difference in hapten density when maleimide-BSA intermediates obtained from 5, 10, 25, 50, 100, and 200 linker:BSA ratios were treated with 25-, 100-, and 400-fold molar excess of MorHap. Subtle differences in hapten density were detected by MALDI-TOF MS at 400 linker:BSA ratio (*p < 0.05 and ****p < 0.0001, two-way ANOVA). Values are the mean of three independent experiments ± standard deviation

The hapten densities that were measured by MALDI-TOF MS were compared to modified Ellman’s test and TNBS assay. Hyperbolic shapes were observed for the three quantification curves indicative of limited attachment sites (Fig. 5a). The quantification curves revealed that the maximum number of haptens that can be attached in BSA was 34–35, which was consistent with the number of surface lysines (30–35) in native BSA [12]. Congruent results also were observed for the modified Ellman’s test and MALDI-TOF MS. These two methods gave the same number of haptens for all the seven MorHap-BSA conjugates and suggested that under optimum reaction conditions, hapten equivalences were similar to hapten density.

The results of TNBS assay were also comparable to MALDI-TOF MS and modified Ellman’s test for MorHap conjugates with 29 and 34 haptens attached. However, the TNBS assay overestimated the number of haptens for MorHap conjugates in the 3–22 hapten range. Interestingly, Adamczyk et al. also observed an overestimation of covalently attached haptens for hapten-BSA conjugates with TNBS [14]. This may be due to conformational changes induced in the protein and/or a masking effect of the nonmodified lysines by the neighboring haptens obstructing the reaction between nonmodified lysines and TNBS. This resulted in an underestimation of the remaining free surface amines on BSA, thereby, giving an overestimation of the number of haptens attached. The overestimation of hapten density by the TNBS assay could be also due to maleimide hydrolysis. Using the modified Ellman’s test, active maleimides were not observed in the MorHap-BSA conjugates, which suggest complete quenching by the hapten or maleimide hydrolysis. The absence of active maleimides in MorHap-BSA conjugates is most likely due to hapten coupling since the maleimide-thiol reaction is significantly faster than the hydrolysis of maleimide to maleamic acid [26].

In terms of the amounts of the sample required and the ease of experimental set-up, MALDI-TOF MS was superior to the two chemical strategies. Since the TNBS assay and the modified Ellman’s test were both colorimetric methods, large amounts of proteins and freshly prepared reagents were needed for reliable results. In contrast, MALDI-TOF MS only requires removal of salts from small amounts of sample by ZipTip. Using MALDI-TOF MS, the modified Ellman’s test and the TNBS assay, the synthesis of reproducible MorHap-BSA conjugates with different hapten densities was established.

### Molar ratio of the hapten is crucial for the preparation of high density MorHap conjugates

Through the use of MALDI-TOF MS, the TNBS assay, the modified Ellman’s test, and the BCA assay, the relationship between hapten density and protein yield was demonstrated. Specifically, the low yield of MorHap-BSA with high density (34–35 haptens) was due to its limited solubility in aqueous buffer. Since the normal function of BSA is to bind fatty acids, it was also possible that the unreacted haptens in solution bound noncovalently to MorHap-BSA conjugates. Thus, the excess unreacted MorHap would be expected to lower the yield of the reaction by further decreasing the solubility of the MorHap-BSA conjugates. The effect of the hapten to maleimide-BSA ratios on protein yield was investigated by treating seven different maleimide to BSA linker ratios with three different hapten ratios: low (25), intermediate (100), and high (400). The protein yield of the resulting 21 MorHap conjugates was determined by BCA (Fig. 5b). When the maleimide-BSA linker ratio of 400 was treated with a 25, 100, and 400 molar conjugation ratio of MorHap, a solution, an opaque mixture, and solid aggregates were observed, respectively. Almost full recovery of the protein was obtained when maleimide-BSA was treated with a low hapten ratio (25:1), 74 % of the protein was recovered with an intermediate ratio (100:1), and approximately 48 % of the protein was rescued at a high hapten ratio (400:1). The protein yields were significantly higher compared to the yield of 5–10 % obtained from the initial unoptimized method. The improvement in yield might be due to the rigorous assays that we employed prior to the coupling of maleimide-BSA to MorHap. Accurate MorHap to maleimide-BSA ratio was achieved by measuring actual protein and thiol amounts by BCA and Ellman’s assay, respectively. The actual MorHap to maleimide-BSA ratio might have been higher than expected in our previous experiments, which results in lower protein yield.

The effect of the hapten to maleimide-BSA ratio (25, 100, and 400) was evaluated in terms of hapten density. Equal portions of the maleimide-BSA intermediate were subsequently treated with 25-, 100-, and 400-molar-fold excess of MorHap to give three MorHap conjugates for each linker ratios (Fig. 5c–i). Analogous to our previous results, the TNBS assay overestimated the results for conjugates with linker to BSA ratios of 5–100 (Fig. 5c–g) while MALDI-TOF MS, modified Ellman’s test, and TNBS assay gave comparable results for the high-hapten-density conjugates (Fig. 5h, i). However, only MALDI-TOF MS was able to discriminate subtle differences in hapten density. MorHap-BSA conjugates derived from maleimide-BSA (34–35 linkers) treated with 100- and 400-fold molar excess of the hapten were statistically increased compared to the MorHap-BSA conjugate that was obtained with 25-fold molar excess of the hapten (Fig. 5i).

Hapten concentration was pivotal for the preparation of BSA conjugates with high hapten density. A low hapten to maleimide-BSA ratio resulted in high protein yield (~100 %), but a lesser number of haptens attached (32). While a high hapten to maleimide-BSA ratio translated to poor protein yield (49 %), but a higher number of haptens attached (34). These data suggest that the intermediate molar conjugation of 100 is the optimal hapten to carrier ratio (74 % yield, 35 haptens attached). In terms of thiol-maleimide coupling chemistry, this corresponds to a 3:1 thiol to maleimide ratio. For the synthesis of MorHap-BSA with varying hapten densities, the optimum procedure involved purification of maleimide-BSA by spin desalting column, purification of the deprotected hapten by petroleum ether washing and subsequent quantification of the thiol concentration by Ellman’s assay, treatment of maleimide-BSA with 100-fold molar excess of the hapten, and purification of the final MorHap-BSA conjugates by dialysis.

### MorHap-BSA conjugates with lower hapten densities bound higher amounts of antibodies

**Fig. 6:**
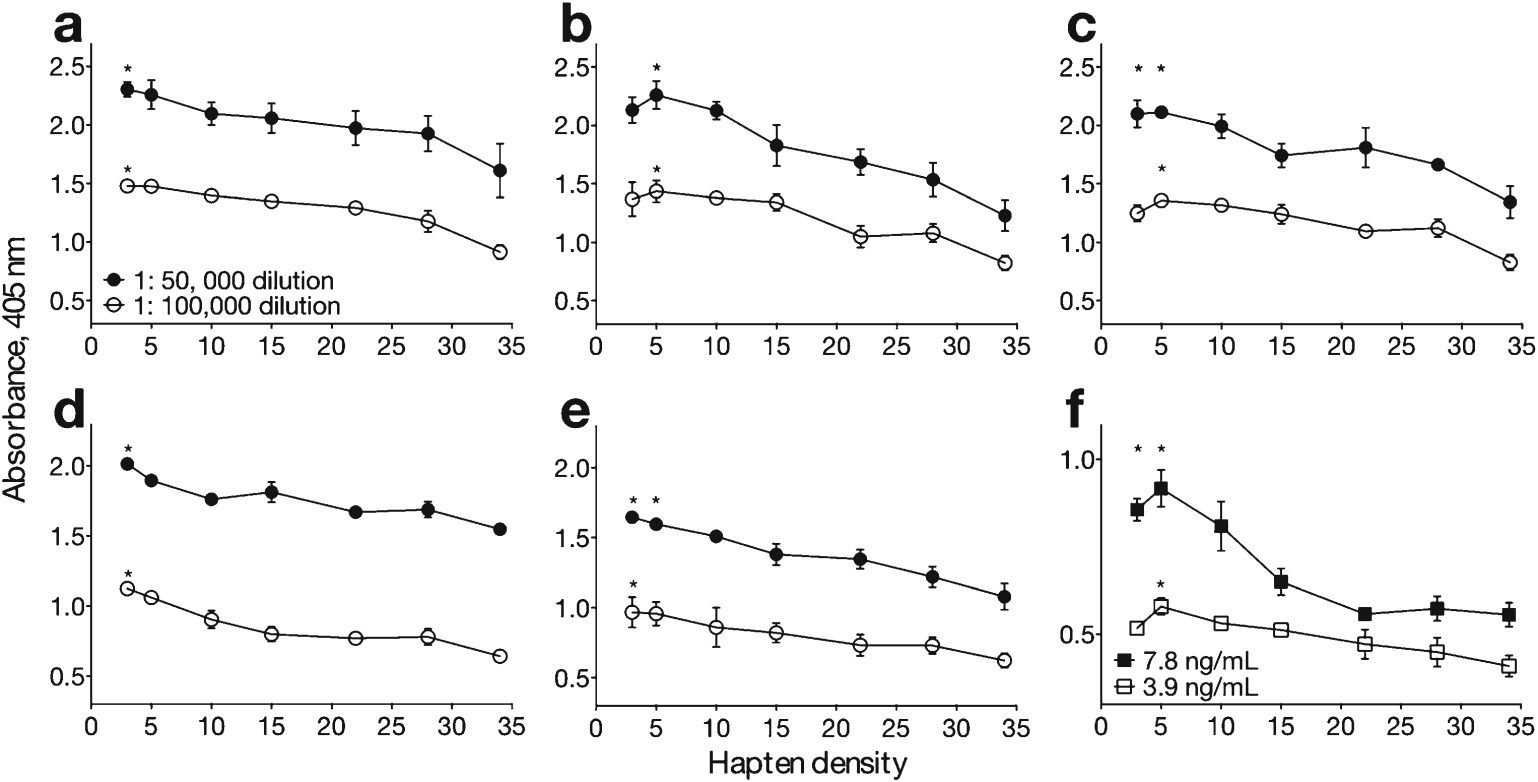
Effect of hapten density on ELISA absorbance. Mouse sera from different animals (**a**–**e**) and monoclonal IgG to morphine (**f**) were assayed against MorHap-BSA conjugates with different hapten densities. Mouse sera dilutions were 1:50,000 (*closed circles*) and 1:100,000 (*open circles*). The *asterisk* indicates that values were significantly different (*p* < 0.05, one-way ANOVA) compared to the highest hapten density (34) group. Values are the mean of triplicate determinations ± standard deviation

Antigen-antibody interactions are crucial in ELISA [28–31]. Antigen-antibody interfaces are primarily controlled by hydrophobic [32] and electrostatic interactions [33], as well as steric effects [28, 34]. MorHap-BSA conjugates with a varying number of haptens were evaluated for their ability to detect antibodies against MorHap [5, 6]. MorHap conjugates with lower hapten densities had higher ELISA absorbances than conjugates saturated with haptens (Fig. 6). These results are consistent with the five mouse sera (Fig. 6a–e) and a monoclonal IgG to morphine (Fig. 6f). ELISA absorbance decreased as hapten density increased at both sera dilutions (1:50,000 and 1:100,000).

We anticipated that BSA with 34 conjugated haptens would give the highest ELISA absorbances because it contains numerous binding sites for the antibody. However, it is possible that the difference in ELISA absorbances was due to the difference in the binding interactions of the MorHap-BSA conjugates to the ELISA plates. Electrostatic effects cannot account for the difference in the ELISA absorbances, since the zeta potential of the conjugates are comparable at physiological pH (Electronic Supplementary Material, Fig. S1). The net charge of the conjugates, which can be correlated to zeta potential, is similar since the depletion of lysine’s positive charge (p*K*_a_ = 10.53) after hapten conjugation is restored by MorHap’s tertiary amine (p*K*_a_ = 8.2). Likewise, the hydrophobicity of MorHap-BSA conjugates cannot explain the difference in ELISA absorbances since the contact angles of the protein conjugates were equivalent (Electronic Supplementary Material, Table S1). There may be small differences in the binding of MorHap-BSA conjugates to the ELISA plates. However, there are no known sensitive assays to detect these small differences.

Steric effects could possibly explain the drop in absorbance with increasing hapten density. A molecule of IgG contains two fragment antigen-binding (Fab) moieties with 4 nm base that are separated by 10–15 nm distance [35–37]. Based on the crystal structure of BSA, a molecule was reported to be ellipsoid with dimensions of 8 × 4 nm [38]. In addition, both the crystal structure and the homology model indicated that surface lysines of BSA were in close proximity [39]. This implies that the surface area of BSA where the haptens were attached is densely packed. This suggests that there is binding penalty to the approaching antibody due to steric effects associated with the “hapten cluster” and antigen-binding site. Based on the above, a hapten-BSA molecule would be expected to accommodate only one Fab moiety of two to five different IgG molecules. The ELISA results indicated that MorHap-BSA conjugates with three to five haptens gave the highest antibody binding and therefore, should be used as the ELISA plate-coating antigen to yield the maximum sensitivity of the assay.

## Conclusion

In summary, this study describes the optimization of a coupling procedure for the synthesis of MorHap-BSA conjugates, which would also be applicable to other thiol-based haptens. The study also demonstrated that MorHap-BSA conjugates with a hapten density gradient could be synthesized in good yields using different molar-fold excess of the linker and optimal hapten:maleimide-BSA molar conjugation ratios. In addition, the number of haptens per molecule of carrier could be accurately measured by MALDI-TOF MS. The modified Ellman’s test and TNBS assay are also useful for determining the number of haptens attached. Using the optimized and quantitative coupling procedure, MorHap-BSA conjugates were produced with high reproducibility. In ELISA, MorHap-BSA conjugates with varying hapten densities were used as coating antigens. The data indicated that lower hapten density conjugates bound higher amounts of antibodies. This suggests that similar hapten conjugates must be prepared with consistency for ELISA sensitivity and the quantification of the haptens bound to the carrier is critical for reproducibility. The synthetic procedures and the analytical techniques that are described in this study will also be useful in the preparation and characterization of other hapten-protein conjugates for vaccine development and assay.

## Electronic supplementary material

Below is the link to the electronic supplementary material.

ESM 1 (PDF 106 KB)
