# Supplementary material for: Characterization and optimization of heroin hapten-BSA conjugates: method development for the synthesis of reproducible hapten-based vaccines
Source: Anal Bioanal Chem. 2014 Aug 2;406(24):5927–37. doi: 10.1007/s00216-014-8035-x (PMC4156789; doi:10.1007/s00216-014-8035-x)
Supplement: Supplementary file 1 — (PDF 106 KB) [file 216_2014_8035_MOESM1_ESM.pdf]

Analytical and Bioanalytical Chemistry

Electronic Supplementary Material

Characterization and optimization of heroin hapten-BSA conjugates: method development for the synthesis of reproducible hapten-based vaccines

Oscar B. Torres, Rashmi Jalah, Kenner C. Rice, Fuying Li, Joshua F. G. Antoline Malliga R. Iyer, Arthur E. Jacobson, Mohamed Nazim Boutaghou, Carl R. Alving, Gary R. Matyas

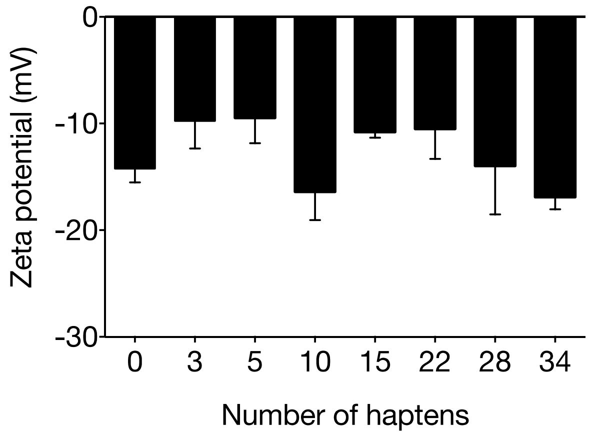

Fig. S1 Zeta potential of BSA and MorHap-BSA conjugates. Zeta potential of the proteins (1 mg/mL, DPBS pH 7.4, 25 °C), was measured using Zetasizer Nano ZS (Malvern Instruments Ltd, Worcestershire, UK). There were no significant differences in the zeta potential of BSA and MorHap-BSA conjugates (one-way ANOVA). Values are the mean of triplicate determinations ± standard deviation.

Table S1 Contact angle of BSA and MorHap-BSA conjugates.

Number of Haptens[a]	Contact angle (degrees)[b]
0	87.7 ± 7.2
3	92.0 ± 5.3
5	87.0 ± 8.0
10	85.0 ± 6.0
15	84.3 ± 1.5
22	90.3 ± 5.0
28	85.0 ± 3.0
34	83.3 ± 0.6

[a] Contact angle of the protein solutions (350 ug/mL, DPBS pH 7.4, 25 °C) was measured against an Immulon™ 2HB flat ELISA plate (Thermo Scientific, Marietta, OH) using the static sessile drop method (Reference S1).
[b] There were no significant differences in the contact angle of the protein solutions (one-way ANOVA). Values are the mean of triplicate determinations ± standard deviation.

Reference S1: Bracco, G., Holst, B. (2013) Surface Science Techniques, Springer Series in Surface Sciences, Vol. 51, Springer-Verlag, Berlin, Heidelberg.
